# A47 A PRELIMINARY ANALYSIS OF THE CROHN'S DISEASE THERAPEUTIC INTERVENTION TRIAL (CD-TDI) IN MILD-TO-MODERATE CROHN'S DISEASE

**DOI:** 10.1093/jcag/gwad061.047

**Published:** 2024-02-14

**Authors:** N Haskey, A Bruno, H Ramay, Y Munazza, L M Taylor, C Ohland, K McCoy, C Ma, S Ghosh, S Veniamin, L Dieleman, M Raman

**Affiliations:** Biology, The University of British Columbia, Vancouver, BC, Canada; National Research Council of Italy, Palermo, Italy; University of Calgary Cumming School of Medicine, Calgary, AB, Canada; University of Calgary Cumming School of Medicine, Calgary, AB, Canada; University of Calgary Cumming School of Medicine, Calgary, AB, Canada; University of Calgary, Calgary, AB, Canada; University of Calgary, Calgary, AB, Canada; University of Calgary Cumming School of Medicine, Calgary, AB, Canada; University College Cork, Cork, Cork, Ireland; University of Alberta, Edmonton, AB, Canada; University of Alberta, Edmonton, AB, Canada; University of Calgary Cumming School of Medicine, Calgary, AB, Canada

## Abstract

**Background:**

Studies show a diet-CD remission link, yet a lack of clinical trials exists. The CD Therapeutic Dietary Intervention (CD-TDI) is an evidence-based dietary regimen based on the principles of the Mediterranean diet and incorporates insights from our pilot data as well as the scientific literature about mitigating inflammation through diet in CD.

**Aims:**

To explore the efficacy of the CD-TDI compared with conventional management (CM) to induce clinical and biochemical remission in patients with active, mild-to-moderate luminal CD.

**Methods:**

Participants (n=28) were randomly assigned (2:1 ratio) to either CD-TDI (n=16) or conventional management (CM) (n=12) for 13 weeks. Stool and blood samples were collected at the start and end of the 13 weeks, and fecal bacteriome was analyzed by 16S rRNA.

**Results:**

At baseline, fecal calprotectin (FCP) or C-reactive protein (CRP) levels were not significantly different. By W13, the CD-TDI group's FCP decreased (△ -113±237 µg/g), whereas the CM group experienced an increase (△ 337±1224 µg/g) (p=0.04). CD-TDI significantly reduced CRP (1.0 mg/L, IQR: 0.6-2.4) compared to the CM (4.5 mg/L, IQR: 2.6-13.0, p=0.007). In the CD-TDI group, zonulin decreased (-43 vs. +26, p=0.04) and increased in the CM group (-43 vs. +26, p=0.04), respectively. Lipopolysaccharide-binding protein (LBP) significantly increased in the CM group compared to CD-TDI (CD-TDI = 17 vs. CM = 26; p=0.03). Differential abundance analysis identified a higher abundance of *Lachnospiraceae, Selenomonadaceae, Sutterella spp.,* and *Akkermansia muciniphila* in the CD-TDI group, whereas *Collinsella intestinalis* was more prevalent in the CM group.

**Conclusions:**

The CD-TDI demonstrated a reduction in markers of inflammation and intestinal permeability in patients with mild-to-moderately active CD, along with alterations in the composition of bacterial taxa. These initial findings strongly indicate that CD-TDI holds promise for alleviating the inflammatory burden in CD patients and warrants further exploration as a valuable adjunctive therapy.

**Funding Agencies:**

Crohn's Colitis Foundation of America; IMAGINE

